# May Salivary Gland Secretory Proteins from Hematophagous Leeches (*Hirudo verbana*) Reach Pharmacologically Relevant Concentrations in the Vertebrate Host?

**DOI:** 10.1371/journal.pone.0073809

**Published:** 2013-09-18

**Authors:** Sarah Lemke, Christian Müller, Elisabeth Lipke, Gabriele Uhl, Jan-Peter Hildebrandt

**Affiliations:** 1 Animal Physiology and Biochemistry, Zoological Institute and Museum, Ernst Moritz Arndt-University, Greifswald, Germany; 2 General Zoology and Zoological Systematics, Zoological Institute and Museum, Ernst Moritz Arndt-University, Greifswald, Germany; Onderstepoort Veterinary Institute, South Africa

## Abstract

Saliva of hematophagous leeches (*Hirudo* sp.) contains bioactive proteins which allow the leech proper feeding and storage of ingested blood, but may also exert effects in the host. Leech therapy is used to treat many different ailments in humans, although only a small fraction of salivary proteins are characterized yet. Moreover, we do not know whether complete transfer of salivary proteins stored in the unicellular salivary glands in a leech to the host during feeding may generate concentrations that are sufficiently high to affect physiological processes in the host. Our 3D reconstruction of a portion of internal leech tissue from histological sections revealed that one leech contains approx. 37,000 salivary gland cells. Using tissue slices from pig liver and mouse skeletal muscle for reference, we obtained data for protein densities in leech salivary gland cells. As individual salivary cells are voluminous (67,000 µm^3^) and the stored proteins are densely packed (approx. 500 µg/mm^3^), we extrapolated that a single leech may contain up to 1.2 mg of salivary proteins. Analyzing protein extracts of unfed or fed leeches by 2D electrophoresis, we calculated the relative molar amounts of individual salivary proteins in the mass range of 17–60 kDa which may be released from a single leech during feeding. Distribution of these salivary proteins in the host (assumed plasma volume of 5 l) may result in concentrations of individual compounds between 3 and 236 pmol/l. Such concentrations seem sufficiently high to exert biochemical interactions with target molecules in the host.

## Introduction

As a consequence of adaptation of leeches to hematophagy, leech salivary gland cells contain a variety of bioactive proteins and peptides [1], [2] which are, at least in part, transferred to the host during feeding [3]. Among other effects, ingredients of leech saliva may suppress inflammation, reduce the intensity of pain and inhibit blood coagulation in the host [4]–[7]. Leeches are extensively used by surgeons to treat venous congestion or hematoma after plastic surgery [7]. Of the many proteinaceous compounds in leech saliva [8], [9], only a few have been identified at the molecular and functional level [1], [10]–[13]. The only one which is well characterized in pharmacological terms is hirudin [6], [14], which functions as a highly effective thrombin inhibitor and prevents clotting of vertebrate blood. Hirudin has been generated in recombinant form, and this has been successfully used for many years in human patients for thrombosis prophylaxis [15], [16].

For only some of the many compounds in leech saliva, the literature reports qualitative information on their potential functions [2], but rarely data about amounts present in the leech, or about effective doses, or about inhibitory constants for physiological effects in the host [17]. In leech therapy, human patients are usually treated with 2 to 6 leeches at a time [5], [7]. Although leech therapy has impressive success rates for very different medical conditions, it is still entirely unclear whether the concentrations of salivary components potentially transferred to the host during feeding may reach effective concentration levels only locally at the wound or also systemically in the host circulatory system.

For this reason, we set out to determine the total amount of stored salivary proteins and peptides that are released by an average unfed leech (*Hirudo verbana*) from salivary gland cells during feeding using histological 3D reconstruction of a small tissue block from body segments containing the salivary gland cells and determination of protein concentrations in individual salivary gland cells. Assuming that most if not all of the secreted material is transferred into the host during feeding, we calculated the concentrations of individual proteinaceous compounds of leech saliva in the host circulation using the staining intensities of individual protein spots as a measure for their contributions to the overall protein mass.

## Materials and Methods

### Animals and Tissue Samples

Medicinal leeches (*Hirudo verbana* Carena 1820) were obtained from Futura Blutegelzucht, Potsdam, Germany, and maintained in glass containers in artificial pond water (0,5 g sea salt dissolved in 1 l deionized water) at 20°C. Fresh pig liver tissue was obtained from a commercial butcher (Greifenfleisch, Greifswald, Germany) and kept on ice until use. One adult female mouse (*Mus musculus,* C57Bl/6) was obtained from the Department of Laboratory Animal Science, University of Greifswald, Germany, and sacrificed by cervical dislocation. Muscle tissue from hind legs was used in this study. Invertebrates (Annelida) are not subject of animal welfare regulations in Germany. Nevertheless, leeches were sacrificed as quickly as possible (fixative or liquid nitrogen) to avoid suffering. Preparation of mouse tissue samples was performed in accordance with the guidelines of the ‘European Convention for the Protection of Vertebrate Animals used for Experimental and other Scientific Purposes’ and the German Animal Protection Act. Permission for keeping the animals and for organ preparation for research purposes was obtained from the State Office for Agriculture, Food Safety and Fisheries of Mecklenburg-Vorpommern, Rostock, Germany.

### Sectioning of Paraffin-embedded Tissue Samples and 3D Modelling

Leeches (n = 5) were stretched out on a perforated wooden rod and fixed in Dubosq-Brasil fixative (1 g picric acid dissolved in 150 ml 80% (v/v) ethanol, 60 ml formaldehyde solution (35%, w/w), 15 ml glacial acetic acid) for 10 min. The anterior body region containing the salivary gland cells was cut off the rest of the body and further fixed in Dubosq-Brasil fixative at 4°C for one week. Subsequently, the teeth at the ridge of the jaws were removed by incubating the samples in dilute nitric acid for 10 h at room temperature. To prevent swelling, the tissue was placed in 250 mmol/l sodium sulfate solution for 24 h and subsequently washed under running water for 24 h. Before being embedded in paraffin, the tissue was dehydrated. To this end, the samples were placed in 80% (v/v) ethanol in distilled water for 2 hours and subsequently for 2×30 min in 96% (v/v) ethanol. Afterwards the samples were transferred to a tetrahydrofuran (THF)-ethanol solution (mixture of equal volumes of THF and 96% ethanol) for 2 h, in pure THF overnight, in THF with paraffin (24 h, 60°C) and finally in pure paraffin (24 h, 60°C).

For embedding, paraffin wax was heated to 60°C. The tissue was placed into a beaker filled with liquid paraffin wax (60°C). The glass panes of a mould were coated with glycerol. The liquid paraffin wax was poured into the mould. Using hot tweezers, the tissue was positioned appropriately into the mould and left under the hood overnight for slow cooling. Histological sections (longitudinal or cross-sections) of embedded leech tissue (6 µm thickness) were prepared on a microtome (Microm HM 360 Rotary Microtome, Thermo Scientific, Dreieich, Germany). Tissue sections were stained using an Azan staining procedure according to Geidies [18].

For 3D reconstruction, 50 Azan-stained serial sections were aligned using AMIRA 4.1 (Visualization Sciences Group, Düsseldorf, Germany). Contours of individual salivary gland cells (total 154) were digitally traced, and a 3D model of each cell in its natural tissue context was generated. Finally, the volumes of the cell bodies were individually calculated using the surface area function of the AMIRA program. The total volume was then used to calculate the natural volume using the voxel/µm^3^ ratio.

### Cryo-sections of Tissue Samples

Leeches (n = 5) were stretched out on a perforated wooden rod and fixed in 5% formaldehyde in phosphate buffered saline (PBS; 0.1 mol/l, pH 7.4) overnight at 4°C. After cutting off the posterior segments, the anterior portion of the leech body containing the salivary gland cells was transferred to a solution of 20% (w/w) sucrose in PBS and incubated for 1 week at 4°C. Tissue was rapidly frozen at −20°C and embedded in Tissue Tek (Sakura Finetek, Zoeterwoude, Netherlands). Cross-sections (6 µm thickness) were prepared using a Leica CM1900 cryostat (Leica Microsystems, Wetzlar, Germany) and transferred to microscopic slides. Tissue samples of mouse skeletal muscle and pig liver, for which weight, volume and protein content [19] had been measured, were used as reference tissues for determining protein density in leech tissues. Sections of all tissue samples (leech, muscle, liver) were mounted onto microscopic slides and simultaneously stained using Bradford reagent [19] for 9 min at room temperature. Coverslips were mounted using Mowiol (Roth, Karlsruhe, Germany). Microscopic inspection and generation of digital images were performed using a Nikon Eclipse TE300 microscope and a Nikon DXM1200 digital camera (Nikon, Düsseldorf, Germany). Densitometric analyses were performed using the density function of a commercial graphics program (Corel Photo Paint).

### 2D Gel-based Semi-quantification of Individual Salivary Proteins

Leeches were prepared for protein extraction after being kept for at least 3 months without feeding (unfed leeches, n = 10) or after voluntary feeding for 30 min on pig blood enclosed in at a piece of pig intestine (fed leeches, n = 4). Each leech was moderately stretched out on a perforated wooden rod and frozen in liquid nitrogen. After 30 s the animal was removed from the rod and a central tissue cylinder (containing the salivary gland cells) was cut from the anterior part of the body. The sample was pulverized using a porcellane mortar upon freezing in liquid nitrogen and subsequently homogenized in lysis buffer (urea 7 mol/l, thiourea 2 mol/l, dithiothreitol 60 mmol/l, CHAPS 30 mmol/l, Biolyte pH 3–10 0.8%, Pefablock 5 mmol/l in A. dest.). After centrifugation for 3 min (4°C, 13,000 g) the sample was aliquoted and the supernatant used for protein determination [19]. Two IEF-strips (Ready Strips™ IPG Strips (Biorad, Munich, Germany), 17 cm, pH 5–8) per sample were incubated with 200 µg protein in rehydration solution (urea 9 mol/l, CHAPS 30 mmol/l, Dithiothreitol 50 mmol/l, Biolyte pH 3–10 0.2% in A. dest.) at 20°C. After rehydration for 12 h, the proteins were separated according to their pI by isoelectric focusing (Protean IEF cell, Biorad, Munich, Germany). IEF-strips were incubated in reducing equilibration buffer (urea 6 mol/l, glycerol 30%, sodium dodecyl sulfate 20%, dithiothreitol 60 mmol/l in Tris 1.5 mol/l, pH 8.8) for 10 min, transferred to jodoacetamide-containing equilibration solution (urea 6 mol/l, glycerol 30%, sodium dodecyl sulfate 20%, jodoacetamide 324.3 mmol/l, Tris 1.5 mol/l, pH 8.8) and incubated for 10 min. Subsequently, the strips were transferred to 2 × SDS sample buffer (Tris 50 mmol/l, glycerol 40%, β-mercaptoethanol 8%, sodium dodecyl sulfate solution 0.2%, bromophenol blue 0,2%) for 5 min. Each strip was then transferred to the top of a 15% polyacrylamide gel (20×20 cm) in a vertical electrophoresis chamber (Biorad, Munich, Germany). Proteins were separated according to molecular masses at 250 V and 20°C for approximately 6 h. After completion, the 2D gels were stained with silver [20]. Spot detection and densitometric analyses were performed using Phoretix 2 D (NonLinear Dynamics, Newcastle upon Tyne, UK).

## Results

### Filling States of Salivary Gland Cells before and after Feeding

**Figure 1:**
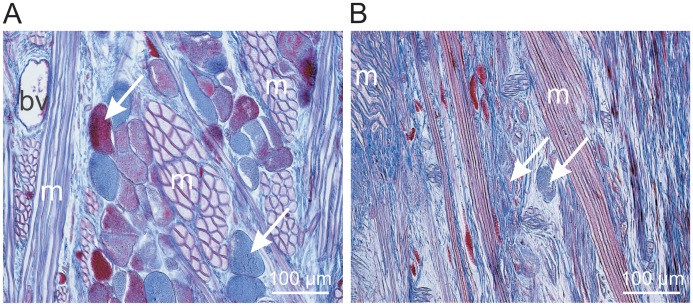
Example images of Azan stained longitudinal sections (6 µm) of leech tissue. Tissue was prepared from the central portion of the anterior body containing the cell bodies of salivary gland cells. A - unfed leech, B - leech fed at a human host for 30 min just before being fixed for histological analysis. The arrows point to individual cell bodies of salivary gland cells. bv -blood vessel, m - strands of muscle cells.

Azan-stained longitudinal sections (6 µm thick) of the paraffin-embedded anterior body part of the leech containing the salivary gland cells were used to compare the filling states of salivary gland cells in unfed (at least 3 months) or fed leeches which were prepared immediately after feeding at a human host for at least 30 min. In unfed leeches, salivary gland cells showed round or slightly elongated cell bodies with diameters of 50–100 µm which were entirely filled with material that stained either blue or red with Azan stain (Fig. 1 A). In tissue sections of fed animals, most of the salivary gland cells appeared much smaller, looked drained and were barely recognizable (Fig. 1 B). These findings indicate that a 30 min feeding period may be sufficient for the leech to mobilize the largest portion of the stored salivary gland proteins and extrude this material to the wound in the host skin.

### Protein Content in Salivary Gland Cells

**Figure 2:**
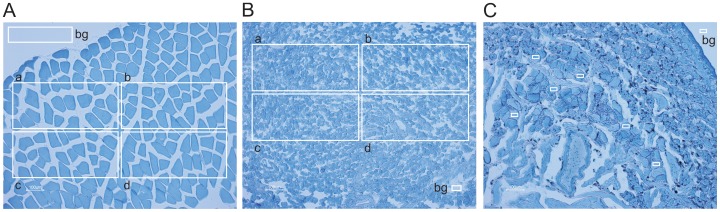
Quantification of protein density in individual salivary gland cell bodies. Mouse skeletal muscle (A) or pig liver (B) were used as reference tissues (for which sample mass, sample volume and protein content had been previously determined) for determining protein densities in leech salivary gland cells (C). Cryo-sections (6 µm) of all three tissue types were mounted onto microscopic slides and simultaneously stained using Bradford reagent. Images were taken using the same settings of microscope and camera. Densitometric analyses were performed on digital images of the reference tissues in the fields a - d. Background (bg) corrected means were used to construct a calibration curve, which was used to read out the protein content of individual cell bodies of leech salivary gland cells using the background corrected density values measured in the fields marked with white squares (C).

To calculate the total protein content in all salivary gland cells in a leech, we determined the average protein concentration in individual salivary gland cells as well as the total number of cells and the total volume of stored material in one leech. Since salivary glands in medicinal leeches are unicellular structures, it was impossible to prepare pure salivary gland cell material for measuring overall protein concentration. Thus, we used an indirect method for determining protein concentration in the storage compartment of salivary gland cells using two types of reference tissues for which weight, volume and protein content (mouse muscle: 109.4±12.3 µg/µl; pig liver: 200.3±9.7 µg/µl, means ± S.D., n = 3) were determined. Cryo-sections (6 µm) of all three tissues were mounted on microscopic slides and simultaneously stained using Bradford reagent. Immediately after staining, the tissue sections were viewed under the microscope and digital images of representative tissue regions were taken (Fig. 2). The mean optical densities of selected fields within the images were measured using the ‘histogram’ function of Corel Photoshop. A two point calibration curve was constructed using the protein content data of the reference tissues as independent parameters on the x-axis and the optical densities of Bradford-stained cryo-sections as the dependent parameters on the y-axis. Using the optical density data of 25 individual salivary gland cell bodies, we calculated the average protein concentration of the storage compartment of salivary gland cells as 476±224 µg/µl (mean ± S.D., n = 25).

### Volume of an Individual Salivary Gland Cell

**Figure 3:**
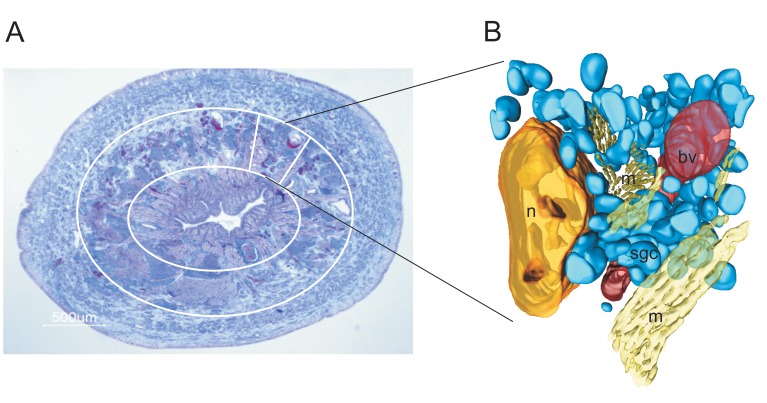
Azan stained cross section through the anterior body of a leech (A) and 3 D reconstruction of a tissue block (B) from a tissue cylinder in this area. The salivary gland cells are located in an area between the gastrointestinal lining (encircled by the inner white circle in A) and the body wall muscle layer (outside of the outer white circle in A). The reconstructed tissue block represents approximately 1/12 of the entire tissue in the circumference of the respective tissue layer. bv - blood vessel, m - strands of muscle fibers, n - nephridium, sgc - salivary gland cell.

To determine the total protein content in the reservoir of an individual salivary gland cell it was necessary to calculate the volume of a single cell. We used digital microscopic images of 50 Azan-stained serial cross-sections (6 µm thickness) of leech tissue that had been embedded in paraffin for 3D modelling (Amira 4.1) of a tissue block of approximately 600×500×300 µm in size. A total of 154 salivary gland cells were reconstructed within this tissue block (Fig. 3). The average volume was 67,048±38,935 µm^3^ per cell reservoir (mean ± S.D., n = 154). Using the data for salivary gland cell protein concentrations as calculated above, we estimated that the protein content within an individual salivary gland cell reservoir was 32±19 ng (mean ± S.D., n = 154).

### Total Number of Salivary Gland Cells

To obtain a reasonable estimate of the total number of salivary gland cells per leech, we fitted the tissue block which had been 3D reconstructed to the circumference and the length of the body segments containing the salivary gland cells. As indicated by the Azan-stained cross section of a leech shown in Fig. 3 A, the presence of salivary gland cell bodies was limited to a tissue cylinder between the thick external layer of body wall muscle and the tissue surrounding the gastrointestinal system. Besides salivary gland cell bodies, the intermediate layer contained nephridia, blood vessels and packages of muscle tissue in different orientations (Fig. 3). The tissue block used for 3 D reconstruction contained 154 salivary gland cells and fitted approximately 12 times in the circumference of the leech body. This accounted for a number of 1,848 salivary gland cells in a cross-sectional body slice of 300 µm thickness. Salivary gland cells are present in the anterior segments of the leech body within segments 5–9 [21]. The mean distance between the anterior front of segment 5 and the posterior border of segment 9 in medium-size leeches is approximately 6 mm. This meant that the number of cells present in one cross-sectional body slice of 300 µm thickness had to be multiplied by 20 to obtain an estimate of the total number of salivary gland cells per leech. Azan-stained serial cross sections of the anterior body of another leech revealed that there was a shallow gradient in the numbers of salivary gland cells from segment 5 to segment 9. As the reconstructed tissue block was prepared from segment 7, we assume that the density of gland cells was in the medium range. Relying on these considerations, the total number of cells in the leech whose tissue block had been used for 3D reconstruction was calculated to 36,960.

### Maximum Concentrations of Leech Salivary Proteins in the Circulation of a Human Host

By multiplying the total number of salivary gland cells per leech with the data for the protein content of individual salivary gland cells, we calculated the total amount of salivary protein per leech which may be released during one round of feeding to 1.2±0.7 mg protein (mean ± S.D., n = 154).

**Figure 4:**
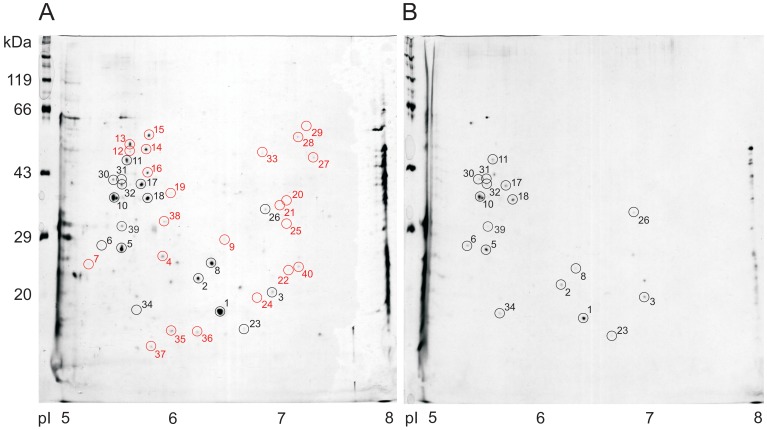
Examples of 2 D gels prepared using protein extracts from the central portions of the anterior body parts of an unfed and a fed leech. A - unfed leech, B - leech fed pig blood for 30 min just before being prepared. Proteins had been separated according to their pI by isoelectric focussing (horizontal axis) and subsequently according to their masses by SDS electrophoresis (vertical axis) and silver stained. Spots labeled by black circles were regularly present in samples from unfed as well as from recently fed leeches, while spots labeled by red circles were generally present in unfed leeches but absent in fed leeches. Proteins represented by the spots labeled in red were considered to be secretory salivary gland proteins which are mobilized during feeding and transferred to the host.

To determine the representation of individual salivary proteins in the entire mass of secretory proteins, we separated proteins extracted from the anterior body of 10 unfed and 4 fed animals (*Hirudo verbana*) on 2D gels (200 µg total protein/gel). Using silver staining, we could identify 40 protein spots which reproducibly occurred in 75% of all samples obtained from unfed animals (Fig. 4 A). Some of these spots could also be observed in 2D gels prepared from protein extracts from freshly fed animals (Fig. 4 B). Densitometric analyses using Phoretix 2D software revealed that many of the spots were less abundant in fed than in unfed animals indicating a certain degree of depletion of these proteins during feeding. Approximately 50% of the reproducible spots in tissue samples from unfed animals, however, were entirely absent in samples of fed animals (Fig. 4, spots labelled in red) indicating that these spots were likely to represent secretory proteins from salivary gland cells that are releasable during feeding. In consequence, further calculations were performed just using the proteins represented by these spots in the gels (Tab. 1). It is unfortunate that there is as yet no annotated database available for genome or relevant transcriptome data of *Hirudo* which made it impossible for us to determine the identities of the protein spots in the gels.

**Table 1:** Relative and absolute protein contents, molecular masses and hypothetical concentrations of individual salivary gland proteins in the circulation of a human host (distribution volume: 5 l).

Proteinspot #	Relative densitometricspot volume (in % ofall proteins)	Absolute amountof protein perleech (µg)	Molecularmass(g/mol)	Absolute molaramount of proteinper leech (mol)	Maximum achievableconcentration in humanplasma (pmol/l)
4	1.6	18.8	27,000	6.9×10^−10^	139
7	2.6	30.7	26,000	1.2×10^−9^	236
9	0.3	3.0	29,000	1.0×10^−10^	21
12	1.9	22.3	51,000	4.4×10^−10^	87
13	3.8	45.4	53,000	8.6×10^−10^	171
14	5.1	60.5	52,000	1.2×10^−9^	233
15	4.0	46.7	58,000	8.1×10^−10^	161
16	2.6	30.4	43,000	7,1×10^−10^	141
19	0.2	2.8	40,500	6.9×10^−11^	14
20	0.2	2.6	38,000	6.9×10^−11^	14
21	0.5	5.3	37,000	1.4×10^−10^	29
22	0.2	2.0	26,000	7.5×10^−11^	15
24	0.3	2.9	20,000	1.5×10^−10^	29
25	0.3	3.8	32,000	1.2×10^−10^	24
27	0.5	5.4	48,000	1.1×10^−10^	22
28	0.7	8.0	54,000	1.5×10^−10^	30
29	0.1	1.6	56,000	2.8×10^−11^	6
33	0.1	0.8	45,000	1.7×10^−11^	3
35	0.4	4.7	17,000	2.7×10^−10^	55
36	0.4	5.0	16,000	3.1×10^−10^	62
37	0.4	5.2	15,000	3.5×10^−10^	69
38	0.4	4.3	32,000	1.3×10^−10^	27
40	0.2	1.9	26,000	7.3×10^−11^	15

We calculated the relative optical density for each spot (Tab. 1, column 1) with respect to the sum of all spots (Tab. 1, column 2). These data were used to calculate the amount of each individual protein (in µg) present in the reservoirs of all salivary gland cells of one leech as a fraction of 1.2±0.7 mg protein per leech (Tab. 1, column 3). From the protein standards used in the second dimension of the 2D analyses, we calculated the molecular masses of individual protein spots (Tab. 1, column 4) and used these data for determining the molar amount of each protein (Tab. 1, column 5). Finally, assuming that the entire protein content of the salivary gland cell reservoirs is released to the wound and flushed away in the blood stream of a potential human host (plasma volume 5 l), we calculated the maximum achievable concentration of each of the protein compounds in human plasma (Tab. 1, column 6). As shown in Tab. 1, the maximum achievable concentrations for these salivary gland cell proteins in the human host were between 3 and 236 pmol/l.

## Discussion

Hematophagous leeches like the medicinal leech *Hirudo verbana* need to feed on body fluids of warm-blooded vertebrates. As they only rarely get the opportunity to feed on proper hosts, they tend to ingest 5–8 times their own body weight of host blood during one meal and store a concentrated suspension of plasma proteins and blood cells in the crop over several months, digesting portions of this material to sustain growth and reproduction [3]. To obtain host blood, the leech has to remain undetected while severing the host’s integument using periodic movements of its saw-like jaws. During uptake of blood and interstitial fluid into the crop, which may take 30 min to 1 h [22], the leech has to prevent clotting of blood cells, inflammatory reactions in and perception of pain by the host. It is assumed that these physiological processes are manipulated by different components of salivary secretions that are released from reservoirs of unicellular salivary gland cells [23], [24] located in the anterior segments of the leech. Elongated portions of the salivary gland cells are connected to small pores located on the rim of the jaws through which the salivary material is directly released into the wound in the host skin [2], [25].

This is, to our knowledge, the first study presenting semi-quantitative estimates about the amounts of salivary gland secretory proteins present in and released by a medicinal leech during a blood meal.

Although structure and functional aspects of salivary gland cells in jawed leeches have been investigated previously [22]–[24], there were only vague estimates about the total number of salivary gland cells. Marshall & Lent [26] calculated that there should be at least 2,000 gland cells present in a single leech. Our results from the 3D reconstruction of a tissue block from the anterior segments of an unfed leech (Fig. 3) are in favor of much higher numbers of salivary gland cells (in this case 36,960). As we have inspected Azan-stained cross sections of different leeches and did not observe obvious individual differences in cell body densities within tissue from the same body region, we assume that the total salivary cell number may rather depend on the size of the animal. Thus, our estimate is that medium size leeches may contain 30,000 to 40,000 salivary gland cells per animal.

The stored material in the reservoir portions of the salivary gland cells seems to be almost completely released during a 30 min feeding period (Fig. 1) which means that a feeding leech may release a total amount of salivary gland protein of approximately 1 mg into the wound. This protein cocktail is comprised of at least 23 different proteins in the molecular mass range of 15 to 60 kDa (Fig. 4) and of proteins and peptides of lower molecular masses not considered in this study. Many of the spots that appear in 2D gels prepared with protein extracts of unfed leeches (Fig. 4 A) are also visible in silver-stained gels prepared with extracts of fed leeches (Fig. 4 B) and belong to the fraction of high abundance proteins. We reasoned that these spots may not be secretory salivary proteins, but represent other types of proteins associated with salivary gland cells or surrounding tissue. To avoid over-estimation of the final concentrations of leech proteins in the host, we excluded all these proteins (labelled in black in Fig. 4 A) from the list of potential secretory salivary proteins. Proteins, however, whose spots were present in extracts of unfed leeches, but not visible in gels prepared with extracts of fed leeches (labelled in red in Fig. 4 A) were assumed to represent secretory salivary proteins.

If these proteins were completely transferred from the feeding leech to the host and would be evenly distributed in the circulatory system of the host, their final concentrations in host plasma would range from 3 to 236 pmol/l. This assumption is likely to be too optimistic since some of the material released to the wound may be readily re-ingested by the feeding leech. Thus, we tried to avoid over-estimation of final concentrations in the host by considering all potential errors resulting in variability of measured parameters in our calculations in the least favorable way. Calculations of final protein concentrations in the host using ‘means minus S.D’-values for protein concentrations in individual salivary gland cells and for spot densities in the 2D gels of unfed animals resulted in final plasma concentrations of individual leech proteins in host plasma in the range between 1 and 103 pmol/l.

Ascenzi et al. [10] analyzed the kinetic parameters of the interaction of hirudin with human thrombin isoforms and reported binding constants (*K* = 8.3×10^13^ mol^−1^ • l, at pH 7.5 and 21°C) that indicate very high affinity between inhibitor and protease. Stone & Hofsteenge [27] reported an inhibitory constant of hirudin for α-thrombin of 2.3 pmol • l^−1^. These data confirm the expectation that long-lasting evolutionary adaptive processes of these parasites to their hosts may have resulted in structurally optimized effector molecules in leech saliva that perfectly fit to specific target molecules in the vertebrate host. Unfortunately, there is only very limited knowledge of kinetic parameters of other leech salivary substances with respect to potential host targets [28]. The data obtained in this study, however, indicate that leech salivary gland proteins transferred to a human host by just one feeding leech may reach concentrations in plasma (Tab. 1) which allow pharmacological effects on local target mechanisms which serve the leech to complete its meal and, in addition, on systemic mechanisms in the host. Thus, leeching may indeed cause prophylactic or therapeutic effects in the host with respect to certain health conditions. Our data support the notion that the reported beneficial effects of leech therapy are not just based on psychological, but rather on pharmacological mechanisms.
